# Exclusive breastfeeding for the first six months of life and its associated factors among children age 6-24 months in Burao district, Somaliland

**DOI:** 10.1186/s13006-020-0252-7

**Published:** 2020-01-30

**Authors:** Abdiasis Jama, Hailay Gebreyesus, Tewolde Wubayehu, Tsigehana Gebregyorgis, Mebrahtu Teweldemedhin, Tesfay Berhe, Negasi Berhe

**Affiliations:** 1 Department of Public Health, College of Health Science, Aksum University, Aksum, Ethiopia; 2 School of Medicine, College of Health Science, Aksum University, Aksum, Ethiopia; 3 Department of Medical Laboratory Sciences, College of Health Science, Aksum University, Aksum, Ethiopia

**Keywords:** Exclusive breastfeeding practice, Children age 6–24 Burao, Somaliland

## Abstract

**Background:**

Adequate nutrition during early childhood ensures growth and development of children and breast milk is better than any other products given to a child. However, studies on exclusive breastfeeding practice are limited in Somaliland. Therefore, this study was aimed to assess exclusive breastfeeding for the first 6 months of life and its associated factors among children 6–24 months of age in Burao district, Somaliland.

**Methods:**

A community-based cross-sectional study was conducted from 26 August to 10 October 2018, in Burao district among randomly, selected 464 mothers with children 6–24 months of age. Data were collected through face-to-face interview using pretested structured questionnaire.

**Results:**

The prevalence of exclusive breastfeeding was 20.47% (95% CI 18.84, 23.63%). Exclusive breastfeeding practice was associated with: having female child (AOR 0.48; 95% CI 0.29, 0.80)), lack of formal education (AOR 0.32; 95% CI 0.19, 0.53), household monthly income 100$-200$ (AOR 0.35;95% CI 0.18, 0.68), lack of husband’s support (AOR 0.32; 95% CI 0.19, 0.53), and mothers who were not counselled on breastfeeding during antenatal care (AOR = 0.33; 95% CI 0.16, 0.66).

**Conclusions:**

Exclusive breastfeeding practice was very low as compared to recommendations of infant and young child practice (IYCF) which recommends children to exclusively breastfeed for the first 6 months of life. Exclusive breastfeeding practice was associated with a mother’s lack of formal education, monthly income less than 100$, being a female child, lack of advice on exclusive breastfeeding during antenatal care and lack of husband support. The promotion of education for women, husband’s engagement, encouraging antenatal care follow-up and counseling of exclusive breastfeeding during antenatal care was recommended to improve exclusive breastfeeding practice.

## Background

According to the definition the World Health Organization (WHO), Exclusive breastfeeding (EBF) is the situation in which an infant receives only breast milk from his/her mother or a wet nurse for the first 6 months and no other solids or liquids with the exception of drops or syrups consisting of vitamins, minerals, supplements, or medicines [1].

Globally, about 40% of infants were exclusively breastfed and this is expected to rise to 50% by 2025. Although the rates of EBF for the past two decades have been increasing, it is still a long road to achieve the 100% global target coverage recommended by UNICEF. This is evident in the current low prevalence of EBF in the developing world particularly in West and Central Africa which happen to have one of the highest rates of infant malnutrition in the world [2, 3].

Despite the WHO recommendations and benefits of EBF, worldwide, only 39% of newborns were put to the breast within 1 h of birth, and only 37% of infants were exclusively breastfed. In Sub-Saharan Africa, 20% of women reported exclusive breastfeeding of their last born infant. In North Africa, the rate of exclusive breastfeeding is 41, 44% in Asia, and the lowest in Latin America at 30% [4, 5].

Overall poor breastfeeding practice, particularly exclusive breastfeeding had been broadly documented in the developing countries and only about 25% of infants in Africa were exclusively breastfed. Additionally, 6% of infants in developing countries were never breastfed [6]. Globally, of 56 million infants, approximately 22 million were exclusively breastfed, while over 34 million children were not. Previous studies showed that 80 % of these children who did not benefit from exclusive breastfeeding in developing countries reside in only 29 countries. From these 29 countries, the 10 large countries including Ethiopia have two-thirds of the approximate numbers of non-exclusively breastfed children [7–9].

In Somaliland, malnutrition is a huge public health problem, negatively affecting the growth, development, and survival of young children. Malnutrition has been aggravated by poor infant and young child feeding practices and other contributing factors including the long-term armed conflict and insecurity, and the breakdown in social and public services. Recurrent droughts and flooding also seriously affecting food security. Existing evidence show that infant feeding practices in Somaliland are alarming and only 13% of the infants have been exclusively breastfed [8]. Exclusive breastfeeding was challenged by a lack of knowledge and different sociocultural beliefs; the incorrect beliefs that breastfeeding mothers are unable to produce enough milk to exclusively breastfeed their child for the first 6 months of life and that society believes that breastfeeding mothers look older than their age. The other barriers include, societal or peer pressure to bottle feed their child. In order to overcome this problem, the Ministry of Health of Somalia and UNICEF has been working together to improve the infant and young child feeding practices.

Exclusive breastfeeding for the first 6 months is one of the infant and young child feeding practices recommended by WHO which can be appropriately assessed by this lifelong EBF practice over time point EBF practice, because the time point EBF practice is mostly assessed by 24 h recall which cannot give us guarantee about the 6 month course EBF practice.

The low prevalence of EBF in most developing countries is attributed to various maternal and child-related factors such as place of residence, sex, age of the child, number of births and space between children, mother working outside home, maternal age and educational level, economic status, mothers’ domestic work burden, access to mass media, maternal healthcare access and use, and maternal knowledge on infant and young child feeding practices [4, 10–16].

There is a paucity of scientific research that investigate the prevalence of EBF and factors affecting EBF in Somaliland, particularly in the study area. Therefore, this study was aimed to determine the exclusive breastfeeding practice for the first 6 months and its associated factors among mothers having children 6–24 months of age in Burao, Somaliland.

## Methods

### Study design and setting

This community-based cross-sectional study was conducted among children aged 6–24 months of age in Burao district, Somaliland, 260 km away from Hargeisa the capital city of Somaliland. The climate of the region is semi-arid and it has a total population of 406,866 [17]. Regarding the health facilities, the city has eight hospitals (two public hospitals and six private hospitals), three health centers, and more than 10 private clinics. The study was conducted from 26 August to 10 October, 2018.

### Sample size and sampling technique

Sample size was determined using single population proportion formula [n = [(Za/2)^2^*P(1-P)]/d^2^] by assuming 95% confidence level of Zα/2 = 1.96, estimated prevalence of exclusive breastfeeding (*P*) as 12.8% [17] from study done in Somaliland, 5% margin of error (d).

The final sample size was 474 mothers. From the five zones in Burao town, two zones were selected by lottery method. Each zone had 12 villages and four villages were selected randomly from each selected zone. The sample size was allocated into the selected eight villages proportionally to the number of mothers with children between 6 and 24 months of age. Systematic random sampling technique was used to select respondent to be interviewed within the selected villages. For those who have had more than two eligible respondents in the same house, lottery method was applied to choose one amongst.

### Data collection tools and quality assurance procedures

Data were collected using structured interviewer administered questionnaire (Additional file 1) which is adopted and modified from different literatures [1, 12, 15, 18]. The questionnaire was first prepared in English and translated to Somali and finally retranslated back to English by a person who can speak both languages. The questionnaire consisted of sociodemographic factors (age, marital status, educational level, monthly income, sex of the child); obstetric and health service related factors (place of delivery, ANC visit, number of ANC visit, postnatal services, colostrum feeding) and psychosocial factors (husband support, family support, knowledge of the mother on EBF). A pretest among 24 (5%) of the sample prior to the actual data collection was carried out in nearby village other than those included in the actual study. Mothers who had 6–24 months old children were asked about their lifelong (6 month course) EBF practice using their recall response. Mothers are categorized as practicing EBF practice if they exclusively breastfeed their child for 6 months, but if they exclusively breastfeed for less than 6 months they are categorized as they don’t practice exclusive breastfeeding. To assess knowledge about EBF, respondents were asked 10 questions on importance of EBF, duration of EBF, contents of breast milk. Respondents that correctly responded > 7 knowledge questions were considered as having good knowledge on EBF, and those who respond < 7 knowledge questions are categorized as having poor knowledge on exclusive breastfeeding. Three trained accountants and five information technicians (IT) collected the data. Three trained public health professionals and the principal investigator supervised the data collection process.

### Data processing and analysis

Data were coded and entered into SPSS Version 21 software for analyses. Descriptive analysis including frequency distribution, proportion and mean was performed to summarize the characteristics of the study subjects. To identify factors associated with exclusive breastfeeding practice, firstly bivariable logistic regression was performed. Subsequently, significant variables in the bivariable analysis (*p* - value < 0.05) were incorporated into the multivariable logistic regression. Statistical significance was declared at *p* < 0.05 and the corresponding 95% CI.

## Results

### Sociodemographic characteristics of the study participants

Four hundred sixty-four mothers who had children between 6 to 24 months of age were included in the study making the response rate of 98%. The mean (± SD) age of the study participants was 27.10 (+ 5.1) years. Two hundred seventy-six (59.5%) of the study participants were in the age range of 25–34 years followed by those in the age range of 15–24 years which accounts for 140 (30.2%). Nearly half of the women 224 (48.2%) had formal education. Two hundred fifty-four (54.7%) of the mothers earned an average monthly income of less than the 246$ (Table 1).

**Table 1:** Sociodemographic characteristics of mothers having children aged 6–24 months from Burao town, Somaliland, 2019 (*n* = 464)

Variable	Category	Frequency	Percentage
Age	15–24	140	30.2
	25–34	276	59.5
	≥ 35	48	10.3
Marital status	Married	401	86.4
	Divorced	46	9.9
	Widowed	17	3.7
Level of education of the mother	No formal education	240	51.7
	Elementary school	106	22.8
	Higher school and above	118	25.4
Occupation status of the mother	House wife	318	68.5
	Merchant	64	13.8
	Private company employed	10	2.2
	Government employed	57	12.3
	Daily labor	15	3.2
Level of education of the father	No formal education	90	19.4
	Elementary school	152	32.8
	Higher school and above	222	47.8
Occupation status of the father	Merchant	125	26.9
	Private company employed	143	30.8
	Government employed	109	23.5
	Daily labor	85	18.3
	Other^a^	2	0.5
Family monthly income	100–200$	185	39.9
	201–400$	172	37.1
	> 400$	107	23.1
Sex of the child	Male	224	48.3
	Female	240	51.7

^a^Other = student

### Obstetric history and health service related factors

Of the 464 mothers who participated in the study, 194 (41.8%) of the mothers had more than three living children at the time of the study. The majority (72.2%) of the mothers had at least one antenatal care (ANC) follow up during their last pregnancy, of which (77%) had four and above ANC visits. Close to one third (28.2%) of the mothers had at least one child death. Close to three-fourth (73.1%) of the mothers gave birth at a health institution. Of the 464 mothers, 79.7% had received advice on breastfeeding (Table 2).

**Table 2:** Obstetric history and health service-related factors of mothers having children aged 6–24 months from Burao town, Somaliland, 2019 (*n* = 464)

Variable	Category	Frequency	Percentage
Age of the mother at first marriage	15–24	415	89.4
	25–34	49	10.6
Age of the mother in first delivery	15–24	355	76.5
	25–34	109	23.5
Parity	1	145	31.2
	2	125	26.9
	3 and above	194	41.8
ANC visit	Yes	335	72.2
	No	129	27.8
Number of ANC visits	Less than 4	77	23
	4 and above	258	77
Receiving information during ANC about exclusive breastfeeding	Yes	246	73.4
	No	43	12.8
	I do not remember	46	13.7
Place of delivery of the baby	Home	126	27.1
	Health facility	338	72.9
Assistant of home delivery	TTBA	93	73.8
	TBA	33	26.2
Postnatal care	Yes	370	79.7
	No	94	20.3
Vaccination of the child	Yes	454	97.8
	No	10	2.2
	Do not know	148	31.9
Screened for HIV	Yes	387	83.4
	No	77	16.6

### Psychosocial factors

Of the 464 mothers who participated in the study, only 163 (35.1%) were receiving support from their husbands. However, 155 (33.4%) of the mothers reported that they received help from other family members. More than half of the mothers 265 (57.1%) had poor knowledge about exclusive breastfeeding (Table 3).

**Table 3:** Exclusive breastfeeding practice among mothers having children aged 6–24 months from Burao town Somaliland 2019 (*n* = 464)

Variable	Category	Frequency	Percentage
Squeeze and discard the first milk	Yes	36	7.8
	No	428	92.2
Reason for squeeze and throw out	To initiate milk production	16	44.4
	Dirty	2	5.6
	Colostrum causes abdominal cramp	18	50
Continuing to breastfeed	Yes	419	90.3
	No	45	9.7

### Exclusive breastfeeding practices

The prevalence of exclusive breastfeeding practice was 20.47% (95% CI 18.84, 23.63%). Almost all the mothers had breastfed at least once in their life time to their youngest child. About 419 (90.3%) of the mothers were still breastfeeding their children during the study period. From all respondents, only 36 (7.8%) of the mothers expressed and discarded their colostrum; 18 (50%) of these mothers who expressed believed that colostrum causes abdominal cramps (Table 4).

**Table 4:** Psychosocial and knowledge factors of mothers having children aged 6–24 months from Burao town Somaliland 2019 (*n* = 464)

Variable	Category	Frequency	Percentage
Help from father	Yes	163	35.1
	No	301	64.9
Type of help	Breastfeeding the child (consultation)	150	92
	Other^*^	13	8
Help from other family	Yes	155	33.4
	No	309	66.6
Breastfeeding knowledge	Good knowledge	199	42.9
	Low knowledge	265	57.1

* Helping workload in the house, preparing and providing food for the lactating mother

### Factors associated with exclusive breastfeeding practices

According to the multivariable logistic regression analysis, educational level of the mother, average family monthly income, sex of the child, ANC visit, and support from husband were identified as statistically significant factors for exclusive breastfeeding practice. Mothers who did not attend formal education and mothers who attended elementary schools were 68 and 52% less likely to exclusively breastfeed their children than those who attended secondary school and above (AOR 0.32; 95% CI 0.19, 0.53 and AOR 0.48; 95% CI 0.29, 0.80).

The average family monthly income was another factor that was found statistically associated with exclusive breastfeeding. Mothers with monthly house hold income of 100$-200$ were 65% less likely to exclusively breastfeed their children than mothers with monthly house hold income of 400$ and above (AOR 0.35; 95% CI 0.18, 0.67). This study revealed that female child was 52% less likely to exclusively breastfeed than male child (AOR 0.48; 95% CI 0.29, 0.80).

Attending an ANC visit was another factor found significantly associated with exclusive breastfeeding practice Mothers who received an ANC visit were 67% less likely to exclusively breastfeed their children than those who had no ANC (AOR 0.33; 95% CI 0.16, 0.66). Additionally, Mothers who did not get support from their husbands were 68% less likely to exclusively breastfeed their children than those who received support from their husband (AOR 0.32; 95% CI 0.19, 0.53) (Table 5).

**Table 5:** Factors associated with excusive breastfeeding among mothers having children aged 6–24 months old in Burao, Somaliland, 2019 (*n* = 464)

Variable	Categories	Exclusive breastfeeding		Odds Ratio (95% CI)	
		Yes	No	Crude Odds Ratio	Adjusted Odds Ratio
Educational level of the mother	No formal education	30 (6.5%)	210 (45.3%)	0.27 (0.13, 0.55)	0.32 (0.19, 0.53)^*^
	Elementary school	25 (5.4%)	81 (17.5%)	0.57 (0.33, 0.97)	0.48 (0.29, 0.80)^*^
	Higher schools and above	40 (8.6%)	78 (17.8%)	1	1
Monthly income	100$-200$	24 (5.2%)	161 (34.7%)	0.28 (0.16, 0.50)	0.35 (0.18, 0.67)^*^
	201$-400$	34 (7.3%)	138 (29.7%)	0.47 (0.27, 0.80)	0.56 (0.30, 1.00)
	> 400$	37 (8%)	70 (15.1%)	1	1
Sex of the child	Male	57 (12.3%)	167 (36%)	1	1
	Female	38 (8.2%)	202 (43.5%)	0.55 (0.35, 0.87)	0.48 (0.29, 0.80)^*^
ANC visit	Yes	84 (18.1%)	251 (54.1%)	1	1
	No	11 (2.4%)	118 (25.4%)	0.27 (0.14, 0.54)	0.33 (0.16, 0.66)^*^
Support from husband	Yes	53 (11.4%)	110 (23.7%)	1	1
	No	42 (9.1%)	259 (55.8%)	0.34 (0.21, 0.53)	0.32 (0.19, 0.53)^*^
Place of birth	Home	16 (3.4%)	110 (23.7%)	0.47 (0.26, 0.85)	0.69 (0.36, 1.31)
	Health facility	79 (17%)	259 (55.8%)	1	1

^*^significant at *p* value < 0.05; Reference Category = 1.00

## Discussion

The prevalence of exclusive breastfeeding in our study is lower than the findings of the Somaliland national policy of exclusive breastfeeding [17], and a study done in Indonesia which shows EBF as 26.2% [19] and 64% in Ghana [20]. Additionally, our finding is lower than the previous study conducted 57.6% in a southern part of Ethiopia [4]. This variation might be due to the socio-economic status of the participants and or access to a health facility and most of the other studies have a lower sample size compared to this study. Study setting difference might contribute to this difference like the study done from Addis Ababa, Ethiopia which was a health facility-based study and could contribute to an increased awareness about EBF practice by the counseling provided during an ANC visit. Age differences between the study subjects might also be a reason because children less than 6 months of age were included in the study from Ghana [20]. Additionally, the low EBF rates in our study might be due to definition of EBF by which this study uses lifelong EBF other than the point time exclusive breastfeeding.

This study revealed that mothers who did not attend formal education and mothers who attended elementary schools were less likely to exclusively breastfeed their children than those who attended secondary school and above. However uneducated mothers exclusively breastfeed their child in a study done in Ethiopia [7]. Another study showed no association between the level of education and practice of exclusive breastfeeding in Somalia [17]. The association found in our study might be due to the role of education in improving awareness about EBF practice and increasing health seeking behaviors like attending an ANC visit.

In this study, mothers with a monthly house hold income of 100$-200$ were 65% less likely to exclusively breastfeed their children than those with monthly house hold income of 400$ and above. A study conducted in Kenya also stated that income has positive association with exclusive breastfeeding [12]. The observed association might be due to the role of income in improving exposure to various media that can improve their knowledge on EBF practice.

Sex of the child was significantly associated with exclusive breastfeeding in this study. A female child was 52% less likely to be exclusively breastfeed than male child. A similar finding was found in a study done in India [21]. However, this finding is not consistent with study done in Nigeria [15]. The association found in this study might be due to the values given for males and females in the community. Mostly, having a baby boy resulted in feeling more pride for the family and due to this, a mother might focus more on baby boys than girls while they feed.

This study indicated that receiving antenatal care was associated with exclusively breastfeed, similar to the positive association observed in Tanzania. This could be due to mothers attending antenatal services being exposed to information about exclusive breastfeeding.

Additionally, support from a husband was associated with exclusive breastfeeding practice. Mothers who did not get support from their husbands were 68% less likely to exclusively breastfeed their children than those who got support from their husband. A study conducted in Hong Kong is in line with the finding of this study [18]. This might be due to the role of husband support in promoting exclusive breastfeeding. As mothers are asked to remember and respond the duration of exclusive breastfeeding retrospectively [22], recall bias is one of the limitations of this study.

## Conclusion

The prevalence of exclusive breastfeeding was very low compared to recommendations of infant and young child practice (IYCF) which recommends children be exclusively breastfed for the first 6 months of life. Mother’s lack of formal education, income less than 100$ per month, being female child, ANC visit, and lack of support from husband were statistically associated with low exclusive breastfeeding practice. Promotion of women’s education, husbands’ engagement, encouraging antenatal care and exclusive breastfeeding counseling during antenatal care were recommended to improve exclusive breastfeeding practice.

## Supplementary information

**Additional file 1.** Questionnaire.

## Data Availability

The datasets on which conclusion was made is available in the form of Microsoft Excel. It is available on reasonable request.

## Abbreviations

ANC: Antenatal Care
EBF: Exclusive Breastfeeding
EDHS: Ethiopian Demographic Health Survey
HEW: Health Extension Workers
HH: House Hold
IYCF: Infancy and Young Children Feeding
MCH: Maternal and Child Health
MPH: Master of Public Health
SNNPR: Southern Nations Nationalities and Peoples’ Region
TBA: Traditional Birth Attendant
TTBA: Trained traditional birth attendant
VIF: Variance Inflation Factor
WHO: World Health Organization
