# Supplementary material for: Exclusive breastfeeding for the first six months of life and its associated factors among children age 6-24 months in Burao district, Somaliland
Source: Int Breastfeed J. 2020 Jan 30;15:5. doi: 10.1186/s13006-020-0252-7 (PMC6993342; doi:10.1186/s13006-020-0252-7)
Supplement: Supplementary file 1 — Additional file 1. Questionnaire. [file 13006_2020_252_MOESM1_ESM.docx]

**Annex 1. Questionnaires to assess exclusive breast-feeding for the first six months of life and its associated factors among children 6- 24 months of age in Burao district, Somaliland**

## **English Version Questionnaires**

## **Part 1: Socio-economic and demographic factors**

| S.NO | Question | Category | Skip |
| --- | --- | --- | --- |
| 1 | What is your age, Mother? | Age in completed year________ |  |
| 2 | What is your marital status, Mother? | 1. Married 2. Divorced 3. Widowed 4. Other(specify) |  |
| 3 | Educational status of the mother? | 1. Illiterate 2. Read and write 3. Primary level 4. Secondary level 5. College and above |  |
| 4 | Occupation status of the mother? | 1. House wife 2. Merchant 3. Private/company employee 4. Government employee 5. Daily labor 6. Others, specify |  |
| 5 | Occupation of the father? | 1. Merchant 2. Private/company employee 3. Government employee 4. Daily labor 5. Others, specify |  |
| 6 | Educational status of the father? | 1. Illiterate 2. Read and write 3. Primary level 4. Secondary level 5. College and above |  |
| 7. | What is your average family monthly income? | Somaliland shillings ___________ |  |
| 8. | Background information of the infant  Sex of the Child? | 1. Male 2. Female |  |
| 1. **Obstetric and gynecologic characteristics of respondents** | | | |
| 9 | What was your age at the time of marriage | Age of marriage _______ |  |
| 10 | What was your age at the time of first birth? | Age of first birth ______ |  |
| 11 | How many children you delivered? |  |  |
| 12 | Did you face child death? | 1. Yes 2. No |  |
| 13 | If question NO12 is yes, how many infant or child was dead? |  |  |
| 1. **Health service related factors** | | | |
| 14 | When you were pregnant to last pregnancy, did you go to the health facility for antenatal care? | 1. Yes 2. No |  |
| 15 | If answer is yes, how many times did you visit? | Number of visits ____ |  |
| 16 | If Q15 is yes, did you receive information about breastfeeding while you were following antenatal care? | 1. Yes  2. No  3. I don’t remember |  |
| 17 | Where you give birth to this baby? | 1. Home 2. Health facility |  |
| 18 | If you deliver at home who assisted you the delivery? | 1. Health professional 2. Trained traditional birth attendant 3. Untrained traditional birth attendant 4. No one |  |
| 19 | Have you ever been informed advice about breastfeeding after delivery? | 1. Yes 2. No |  |
| 20 | If 19is yes, about which of the following characteristics you have been informed or advised regarding breastfeeding?  Multiple response is possible | 1. Initiate breast feeding immediately after birth 2. No pre-lacteal feeding to be given 3. Breast feeding babies less than six months do not require extra food even water. 4. Continue breast feeding during illness of the baby |  |
| 21 | Have you vaccinated your child? | 1. Yes 2. No |  |
| 22 | If Q21is yes do you remember the kind of vaccine(s) they have taken? |  |  |
| **IV. Questions about Knowledge of respondents on Exclusive breast feeding practice** | | | |
| 23 | Is exclusive breast feeding mandatory for the growth of a child? | 1.Yes  2.No |  |
| 24 | When do you think a child is exclusively breast feed? |  |  |
| 25 | How soon after child birth should  Breast feeding was started? |  |  |
| 26 | How frequent should a child be breastfeed per day? |  |  |
| 27 | What other additional foods or drinks Should a child be fed from birth up to six months in addition to breast milk? | 1. Nothing 2. Plain water 3. Water-sugar/salt solutions 4. Cow’s milk 5. Formula milk 6. Butter   7.Other, specify _________ |  |
| 28 | When should a child be supplemented with additional complementary foods? | At (___________] months |  |
| 29 | How long in total should a child be breastfeed? In months | (_______] months |  |
| 30 | What is the importance of exclusive breast feeding to the mother? |  |  |
| 31 | Do you believe that formula feeding can replace breastfeeding? | 1.Yes  2.No |  |
| 32 | What are the consequences of not breastfeeding your child? |  |  |
| 33 | What is the importance of colostrums to your child? | 1._____________________  2. I don’t know |  |
| 1. **Exclusive breastfeeding practice of respondents** | | | |
| 34 | Did you squeeze and throw out the first milk (colostrum)? | 1. Yes 2. No |  |
| 35 | If answer is yes, what was the reason? | 1. To initiate milk production 2. Dirty 3. Colostrum causes abdominal cramp 4. Other specify |  |
| 36 | Are you still breastfeeding the child? | 1. Yes 2. No |  |
| 37 | When you usually breast feed your youngest child?  (more than one answer is possible) | 1. On the demand 2. When the child cries 3. On schedule 4. On convenience 5. In other condition (specify |  |
| 38 | For how many months did you feed with breast milk only? | I feed for ________ months breast milk only |  |
| V Psychosocial factor | | | |
| 39 | Do you get any help about breastfeeding from your husband? | 1. Yes 2. No |  |
| 40 | If Question NO45, What kind of help he gave you? |  |  |
| 41 | Is there any one from the family who helped to breastfed your child? | 1. Yes 2. No |  |
| 42 | What kind of help did he/she give you? |  |  |
